# Transforming dental diagnostics with artificial intelligence: advanced integration of ChatGPT and large language models for patient care

**DOI:** 10.3389/fdmed.2024.1456208

**Published:** 2025-01-06

**Authors:** Masoumeh Farhadi Nia, Mohsen Ahmadi, Elyas Irankhah

**Affiliations:** ^1^ Department of Electrical and Computer Engineering, University of Massachusetts Lowell, Lowell, MA, United States; ^2^ Department of Electrical Engineering and Computer Science, Florida Atlantic University, Boca Raton, FL, United States; ^3^ Department of Industrial Engineering, Urmia University of Technology, Urmia, Iran; ^4^ Department of Mechanical Engineering, University of Massachusetts Lowell, Lowell, MA, United States

**Keywords:** dental, diagnosis, ChatGPT, artificial intelligence, LLM, NLP, patient care

## Abstract

Artificial intelligence has dramatically reshaped our interaction with digital technologies, ushering in an era where advancements in AI algorithms and Large Language Models (LLMs) have natural language processing (NLP) systems like ChatGPT. This study delves into the impact of cutting-edge LLMs, notably OpenAI's ChatGPT, on medical diagnostics, with a keen focus on the dental sector. Leveraging publicly accessible datasets, these models augment the diagnostic capabilities of medical professionals, streamline communication between patients and healthcare providers, and enhance the efficiency of clinical procedures. The advent of ChatGPT-4 is poised to make substantial inroads into dental practices, especially in the realm of oral surgery. This paper sheds light on the current landscape and explores potential future research directions in the burgeoning field of LLMs, offering valuable insights for both practitioners and developers. Furthermore, it critically assesses the broad implications and challenges within various sectors, including academia and healthcare, thus mapping out an overview of AI's role in transforming dental diagnostics for enhanced patient care.

## 1 Introduction

Artificial intelligence is expected to revolutionize sectors such as healthcare and dentistry by presenting new approaches to various clinical problems. This would enhance the productivity of medical practitioners. The ChatGPT model has been developed by OpenAI using the GPT [Generative Pretrained Transformer) framework, a deep learning model that is utilized in natural language processing (NLP)] (1). The model can produce text that is like human writing and can interact with users via chat platforms. With ChatGPT's generative AI, original content can be generated in real-time discussions (2, 3). A variety of artificial intelligence models are employed to provide conversational responses to inquiries based on extensive text data. ChatGPT is designed to remember both user input and its own responses within a conversation, allowing it to build upon previous responses as new inquiries are received (4). The effectiveness of ChatGPT has been considered in several healthcare domains, including its capacity to diagnose dental problems. It has been demonstrated that it provides accurate differential diagnoses in a substantial number of cases, compared favorably with the diagnostic abilities of healthcare professionals (5).

In the realm of dentistry, research on ChatGPT's effectiveness remains limited. There has been research on ChatGPT's capability to produce scientific content within oral and maxillofacial surgery, as well as its accuracy and dependability in providing concise clinical responses in endodontics (6–8). In addition, its proficiency has been assessed in various scenarios, including board-style dental knowledge quizzes, and answering questions related to scientific or research writing. Health care and dentistry will benefit greatly from the deployment of ChatGPT because it will increase patient autonomy, enhance the efficiency and safety of services, boost sustainability, and broaden the range and quality of care, ultimately empowering patients (9). Artificial intelligence finds numerous applications in prosthodontics, such as implant-supported prosthetics, computer-aided design (CAD), maxillofacial prosthetics, computer-aided manufacturing (CAM), and both fixed and removable prosthetics (10). Research on ChatGPT's application within medical and dental disciplines lacks extensive systematic reviews and meta-analyses. The utility of ChatGPT has been examined in numerous healthcare contexts including dental, However, these studies often have limited scope and fail to provide insights into the potential benefits and drawbacks of integrating ChatGPT into these fields., often consisting of only literature reviews and editorials (11–13). It is anticipated that ChatGPT will enhance the creation of precise and documentation, facilitating collaboration and knowledge exchange among oral medicine practitioners across clinics, hospitals, and departments (12). Oral medicine professionals can utilize ChatGPT's text-generation features to input patient data, clinical observations, and treatment strategies, enabling them to create detailed reports. This not only enhances the quality of documentation but also facilitates effective communication among healthcare providers. Moreover, oral medicine specialists can utilize ChatGPT for virtual patient interactions, offering advice, responding to inquiries, and discussing treatment options. It also assists in the decision-making process in cases involving complex oral medicine. The primary objective of this review was to provide a detailed, evidence-based evaluation of ChatGPT's potential as a resource for medical and dental research, with the goal of directing future investigations and informing clinical practice.

## 2 AI and data analysis in dentistry

**Figure 1:**
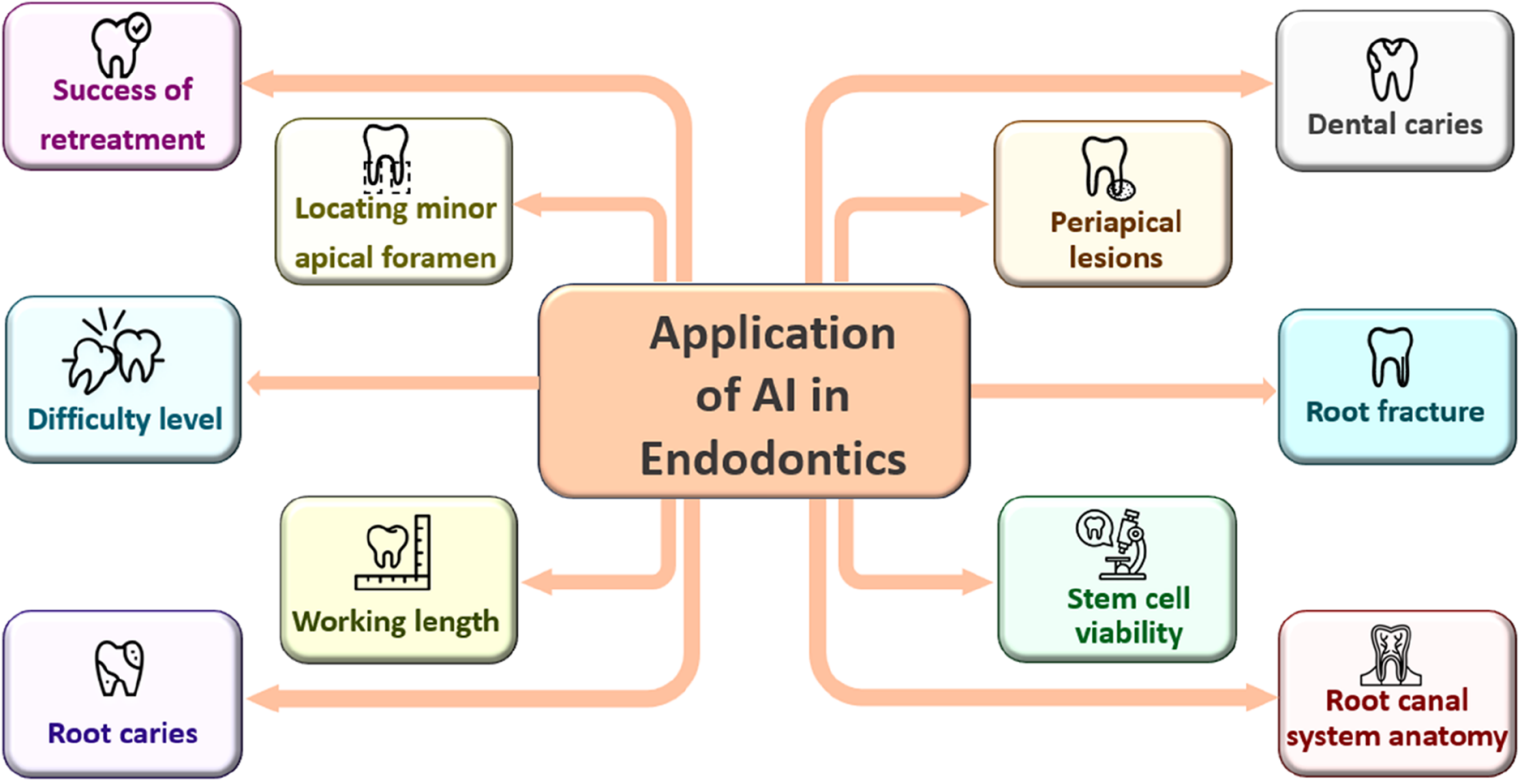
The multifaceted effects of artificial intelligence on endodontic practice (19).

Within the medical community, ChatGPT is gaining recognition for its influence on both the healthcare system and medical field. It provides support as a complementary resource for diagnosis and decision-making across a variety of medical disciplines (14). Although the accuracy of its outputs and the potential for reinforcing biased diagnoses have prompted discussions on the necessity of human oversight when using this technology, ChatGPT can also be utilized in assessing disease risks and outcomes, advancing drug development, enhancing biomedical research, and transforming healthcare practice in significant ways (15–17). Artificial intelligence has made remarkable progress in the field of digital health, particularly in dentistry. In addition to diagnosing dental issues through the analysis of imaging, it also aids in the planning of medical operations (18). The capabilities of artificial intelligence extend to the analysis of auditory data to improve understanding of oral functions, thereby supporting dental education. The potential applications of AI in endodontics are demonstrated in Figure 1.

**Figure 2:**
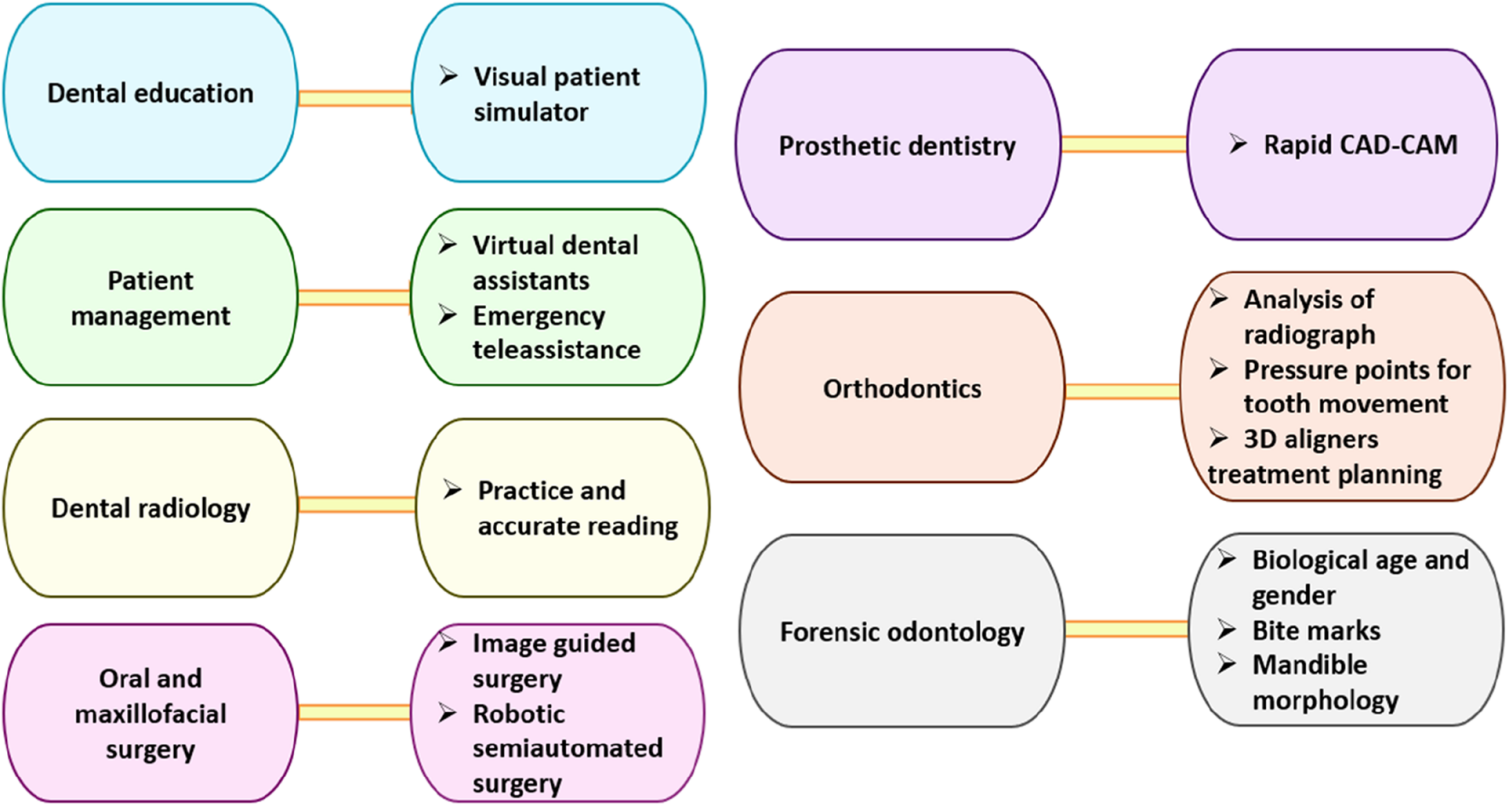
Diverse applications of AI in dental specialties, including dental education, patient management, radiology, surgery, prosthetic dentistry, orthodontics, and forensic odontology, by enhancing training, efficiency, accuracy, and patient care.

As evidenced by a bibliometric analysis showing an increase in publications on the subject from a single article in 2000 to 120 in 2023, with a peak of 208 articles in 2022, AI's applications within dentistry are on the rise (20). The use of neural networks in diagnosing endodontic problems has been proven to be as accurate as that of dental professionals, offering benefits to nonspecialists and those new to the field (21). Studies have shown the wide-ranging potential of artificial intelligence in endodontics, emphasizing how it can enhance diagnostic precision, treatment planning, and educational outcomes. Figure 2 illustrates how artificial intelligence (AI) is revolutionizing dental practice across different specialties. Visual patient simulators are used in dental education to enhance training experiences. With the help of virtual dental assistants and emergency teleassistance, patient management can be more efficient and accessible. By using AI, dental radiologists can read and interpret radiographs more accurately, enhancing diagnostic accuracy. Surgical accuracy and outcomes are improved through the use of AI in oral and maxillofacial surgery.

Artificial intelligence is used in rapid CAD-CAM systems to streamline dental prosthetic design and production. The use of AI in orthodontics allows for the analysis of radiographs, the optimization of tooth movement, and the planning of 3D aligner treatments. Furthermore, forensic odontology employs AI for determining biological age and gender, analyzing bite marks, and assessing mandible morphology to assist with forensic investigations. AI is having a profound impact across a wide range of dental specialties, improving both clinical outcomes and patient care. Mohan et al. (22) assert that despite concerns regarding patient privacy and potential misinterpretations, the integration of AI-driven care systems in dentistry will improve patient care, encourage innovative research, and require collaboration across various professional fields in the future. Additionally, OpenAI's Generative Pre-Trained Transformer, GPT-4, represents an advancement in AI language technologies, impacting dentistry and healthcare profoundly (23).

Mohamed M. Meghil et al. (24) present a systematic review adhering to PRISMA standards and the Cochrane Handbook, analyzing the evolution and integration of artificial intelligence (AI) in various dental specialties. Their research encompasses 28 studies from distinct fields such as pathology, maxillofacial surgery, and orthodontics, among others, sourced from PubMed and Web of Science. These studies were evaluated for methodological quality and bias with the PROBAST tool, highlighting AI's role in clinical data analysis across dentistry. The findings underline the substantial datasets available for training and testing, indicating AI's promise for improving patient outcomes, diagnostic processes, and treatment planning. However, the authors advocate for additional research, specifically randomized clinical trials, to solidify AI's efficacy in dental practice, aiming for data-driven, superior dental care that revolutionizes treatment delivery. Thilagar et al. (25) explored the link between acute myocardial infarction (AMI) and periodontal disease (PD), focusing on the shared systemic inflammation indicated by C-reactive protein (CRP) levels. They emphasized the overlap in the production of proinflammatory and anti-inflammatory cytokines, signaling molecules, and enzymes in both conditions, which contribute to their chronic inflammatory nature. The study employed machine learning to identify key hub biomarker genes involved in the interaction between AMI and PD. Eggmann et al. (26) provided an overview of the implications of ChatGPT and other LLMs for dental medicine. The article explores these implications, noting that while ChatGPT is effective in language-related tasks, it has limitations like producing incorrect content and misinformation. The direct impact on dental practitioners is minimal, but LLMs could influence administrative tasks and telemedicine, offering benefits such as clinical decision support and text summarization.

The review by (20) provides a historical overview of AI, from its conceptual inception in 1950 to its current applications in dentistry, with particular attention on diagnostic imaging and computational challenges. Marta Revilla-León et al. (27) conduct a systematic review of AI applications in implant dentistry, specifically focusing on design optimization, implant type identification, and success prediction. The research suggests that AI has promising accuracy for implant recognition and highlights areas for improvement, particularly in success prediction models. Another study (28) examines AI's impact on dental imaging, showcasing its ability to improve diagnostic accuracy, speed, and overall patient care. Despite advancements, more clinical trials are required to address limitations in AI-based imaging. The research by (29) highlights the gap in AI adoption in dentistry within low- and middle-income countries, emphasizing a need for inclusive studies to expand AI's benefits globally.

The evolution of language modeling has paved the way for sophisticated frameworks such as GPT, BERT, and ELMo. ChatGPT, as an advanced Large Language Model (LLM), demonstrates strong conversational abilities and potential for dental applications. The study by (23) explores the use of LLMs in dentistry, focusing on automated dental diagnosis and cross-modal approaches. LLMs like ChatGPT are employed for text mining of medical records, natural language reasoning for treatment planning, and NLP for medical documentation. Cross-modal dental diagnosis leverages multimodal data, using visual and auditory information for enhanced diagnostics. The research also addresses the challenges of incorporating LLMs in dentistry, such as data quality, model bias, privacy concerns, and computational limitations. It suggests human oversight to correct potential inaccuracies, emphasizes neural-symbolic models to mitigate bias, and underscores strict data privacy protocols to enhance AI adoption in dental practice.

ChatGPT, a member of OpenAI's GPT (Generative Pre-trained Transformer) family, is based on transformer architecture, which is key to its deep learning capabilities. The model ranges from 12 to 96 transformer layers with multiple attention heads per layer, allowing it to focus on different parts of the input sequence. Embedding dimensions range from 768 to 12,288 depending on the model scale, contributing to the richness of text representations. GPT-3, for example, contains 175 billion parameters, enabling it to generate coherent and contextually relevant text. ChatGPT is trained using a mix of publicly available text and licensed datasets, initially with unsupervised learning, followed by fine-tuning for human-like responses (30). The latest iteration, GPT-4, introduces significant advances in the medical field, including tools such as an Advanced Image Generator, Code Interpreter for data processing, and a Browser Tool for accessing up-to-date information. It also integrates DALL·E for generating visuals and a Text-to-Speech tool for spoken output. These features make GPT-4 a powerful assistant for healthcare professionals, enabling tasks like dental diagnostics and patient education (31, 32). To enhance data management and improve diagnostic precision in dentistry, ChatGPT can be integrated with Electronic Dental Records (EDRs). Patel et al. (33) examined EDRs for classifying patients’ smoking status using machine learning classifiers, finding that support vector machines were most effective. This demonstrates how EDRs can provide detailed patient insights, which is often overlooked in traditional Electronic Health Records (EHRs).

The use of EDRs is limited to secondary purposes, such as conducting research or analyzing treatment outcomes. In addition (34), discusses both the challenges and opportunities associated with leveraging EDRs for dental research. There is a prevalent use of unstructured text in EDRs, which makes their use in research or outcome analysis difficult. While NLP techniques are acknowledged for their ability to extract detailed information from clinical notes in EDRs, the paper highlights obstacles to their widespread adoption. Among these challenges are the lack of applications of NLP in primary oral healthcare, the difficulty of generalizing findings due to dentistry's unique vocabularies, and the difficulties associated with cross-referencing results and validating them. An overview of ChatGPT and EDRs is shown in Table 1.

**Table 1:** An overview of ChatGPT and electronic dental records (EDRs).

Researchers	Techniques	Data quantity	LLM categories	Inquiry technique	Evaluation criteria
Mago and Sharma (35)	Interrogating an LLM about imaging features, oral anatomy, and disease	Set of 80 queries	Conversational Model GPT-3	Unprompted	4-tier modified preference scale
Chuang et al. (36)	Use of GPT-J for prompt generation in NER models for diagnosis extraction from electronic dental records	Dataset of 5,495 eligible patients, resulting in 8,125 clinical notes for periodontal diagnosis extraction	GPT-J, RoBERTa with spaCy package	Direct testing and seed generation for feeding NER models	Performance measured by F1 score, highlighting the importance of seed quality. Consistent performance across settings with F1 scores between 0.92-0.97 after RoBERTa training
Doshi et al. (37)	Refinement of imaging summaries	254 imaging summaries	Conversational Models GPT-3.5, GPT-4, Bing, and Bard	Varied query structures	Comprehensibility metrics
Mykhalko et al. (2023) (38)	Evaluating diagnostic capabilities using ChatGPT-3.5 with various chat setups	50 clinical cases	ChatGPT-3.5	Three-phase experiment with different information and prompt setups	Diagnostic accuracy percentages across phases (66%, 70.59%, and 46%), highlighting the strength of ChatGPT in structured scenarios and the importance of prompt engineering.
Lai et al. (39)	Evaluating ChatGPT-4's performance on the United Kingdom Medical Licensing Assessment (UKMLA)	191 SBA questions from UKMLA	ChatGPT-4	Three attempts over three weeks with structured SBA questions	Average performance score of 76.3% across three attempts, with analysis on areas of both strength and weakness in specific medical fields. Consistency in correct and incorrect responses evaluated
Jeblick et al. (40)	Condensing imaging narratives	Trio of imaging narratives	Conversational Model GPT	Singular query structure	5-tier preference scale
Lyu et al. (41)	Converting imaging narratives into layman's terms	62 thoracic CT and 76 cranial MRI summaries	Conversational Model GPT-4	Multiple query structures	5-tier preference scale
Waters et al. (42)	Practical applications and suggestions for interacting with LLMs like ChatGPT in radiation oncology	Not specified	ChatGPT	Guide on how to interact with LLMs in clinical and administrative tasks	Highlights the potential uses, limitations, and ethical considerations of LLMs in radiation oncology, emphasizing the importance of human review
Chiesa-Estomba et al. (43)	Evaluating Chat-GPT as an aiding tool for sialendoscopy clinical decision-making and patient information support.	Not specified	ChatGPT	Prospective, cross-sectional study comparing Chat-GPT with expert sialendoscopists	The study compared Chat-GPT and expert sialendoscopists’ agreement on salivary gland disorder management (Chat-GPT: 3.4, experts: 4.1) and evaluated therapeutic alternatives suggested by both

The study (44) explored ChatGPT's proficiency in answering frequently asked questions (FAQs) regarding fluoride based on the American Dental Association (ADA) guidelines. Using structured methodology, ChatGPT was asked a series of eight specific fluoride-related questions on two separate occasions, May 8th and May 16th, 2023. The recorded responses from ChatGPT were compared with those from the ADA's website. ChatGPT's responses and those provided by the American Dental Association were analyzed primarily qualitatively to identify similarities in wording and context. In this assessment, ChatGPT's answers were compared with the official ADA answers to assess their consistency over the week-long period. While ChatGPT's responses were consistent over time, they were more detailed and scientific than those of the ADA, even though both fundamentally conveyed the same information about the role of fluoride in dental health. In addition to radical dental information dissemination, these studies present promising applications in radiology for more efficient and accurate medical image diagnoses, which could result in improved patient care and lower healthcare costs. AI technology in clinical applications has significantly enhanced patient outcomes, streamlined processes, and reduced costs. In dentistry, convolutional neural networks have demonstrated improved accuracy in identifying and classifying maxillofacial fractures, highlighting AI's transformative potential in dental diagnostics. Notably, ChatGPT's accuracy in various dental and surgical domains, including oral and maxillofacial surgery, has been studied (10, 45).

For example, GPT-3 was found to accurately identify anatomical points in oral radiography with 100% accuracy, though Mago et al. (35) cautioned against relying solely on it due to occasional inaccuracies. Similarly, Vaira et al. (46) conducted a study in August 2023 to evaluate ChatGPT's accuracy in head and neck oral maxillofacial surgery, finding that 87.2% of the responses were accurate and 73% were comprehensive. Despite these promising results, researchers noted that ChatGPT is not fully reliable for decision-making in complex surgical cases. In prosthodontics, it is crucial to assess the accuracy and consistency of ChatGPT. Such an assessment involves comparing ChatGPT's responses to established knowledge and guidelines in prosthodontics. Consistency is measured by checking if the AI provides similar answers to repeated or rephrased questions. Currently, there is a lack of literature on the application of ChatGPT in interceptive orthodontics.

**Figure 3:**
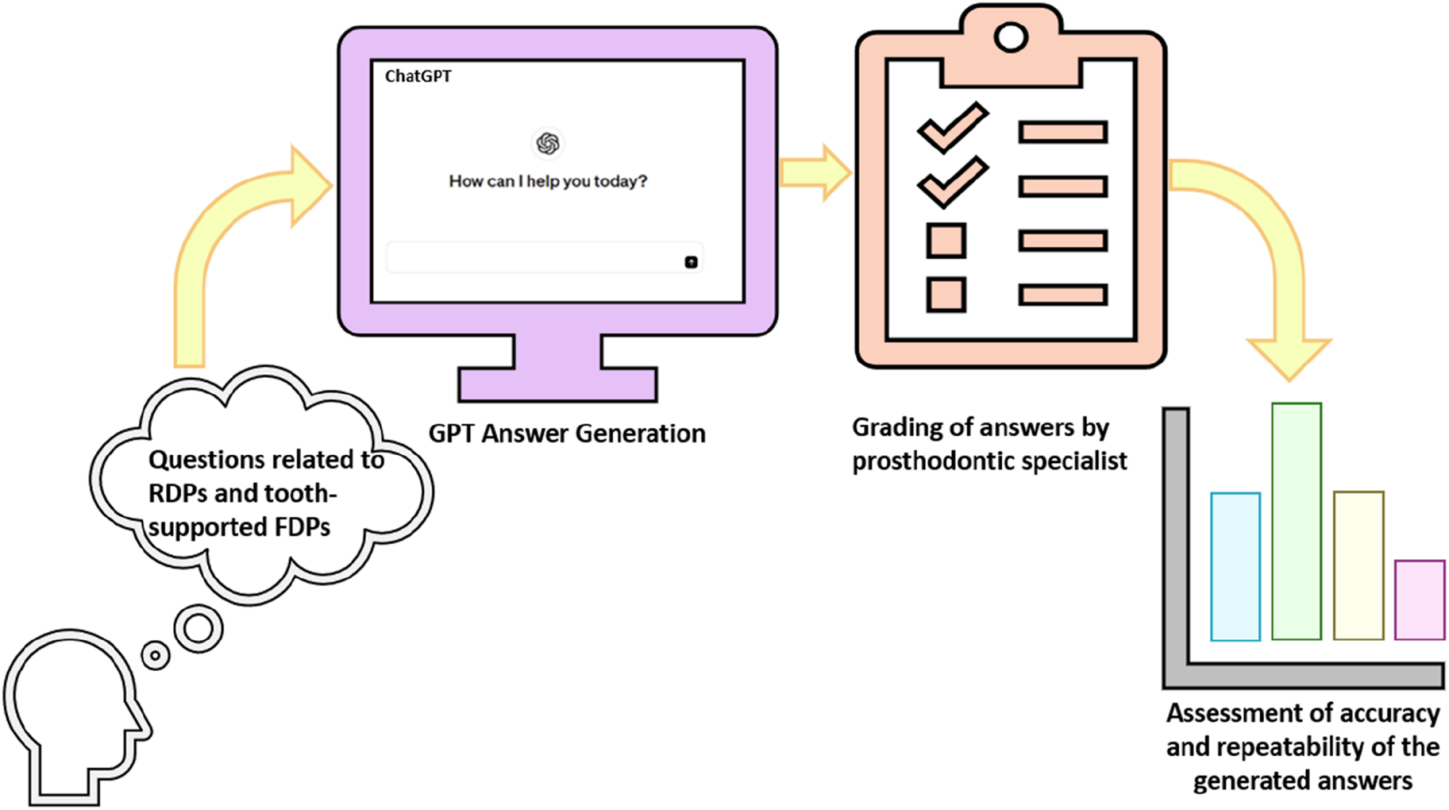
The approach employed to assess the accuracy and consistency of responses generated by ChatGPT to questions regarding RDPs and tooth supported FDPs.

In the following Figure 3 outlines the flowchart for assessing ChatGPT's accuracy and consistency in responses related to Removable Dental Prostheses (RDPs) and Fixed Dental Prostheses (FDPs). The process starts with formulating questions about these dental topics and analyzing ChatGPT's responses.

The questions are then entered into the ChatGPT interface, which begins the process of generating GPT answers. A prosthodontic specialist grades the responses obtained from ChatGPT based on their accuracy and relevance (47, 48). As a final step in the methodology, the graded answers are statistically analyzed to determine both the accuracy of the ChatGPT answer and the repeatability of the answers across multiple interactions. In the field of orthodontics, Subramanian et al. (49) demonstrate AI's potential to simplify cephalometric tracing for everyday clinical use and to conduct extensive data analysis for research. In a study by Tanaka et al. (50), they found that ChatGPT was effective in delivering informative responses on topics related to orthodontics, such as digital imaging, clear aligners, and temporary anchorage devices. Tanaka et al.'s study is similar to the current study but differs in those ten orthodontists with specialized training from various Italian orthodontic schools evaluated the patients instead of five general orthodontists as in Tanaka et al.'s study. Duran et al. (51) acknowledge that while ChatGPT can provide reliable information on cleft lip and palate, its complex responses need professional verification. The AI tool, aware of its limitations, advises consulting a specialist for orthodontic decisions. Despite its usefulness, ChatGPT cannot replace the essential in-person aspects of dental care. However, it could streamline administrative tasks in dental practices, such as managing insurance claims and supporting dental telemedicine. Gonzales et al. (52) suggest that AI can match expert human observers in determining cervical vertebral maturity stages, potentially increasing diagnostic precision, and improving orthodontists’ efficiency. The authors of this study hold a different view, noting that the orthodontists involved did not find the ChatGPT responses completely accurate. For both open-ended and clinical case questions, accuracy scored 4.9 out of 6, while completeness scored 54.3%. Furthermore, Ahmed et al. (53) have shown that AI can develop precise caries detection models, aiding clinical decision-making, and improving patient care. AI has not yet substantially improved the ability to provide answers to open-ended questions, diagnoses, or treatment planning for clinical cases, despite x-ray analysis being a static process.

## 3 AI-based systems developed and medical training

AI in healthcare is often developed by computer scientists without medical training, leading to a focus on technical problem-solving rather than the holistic care provided by clinicians (54). Despite AI's advantages in dentistry, such as improving speed, accuracy, and standardization, adoption is slow among dental professionals due to steep learning curves, high costs, and extensive data requirements (55). While AI has proven useful in fields like medical imaging, such as identifying COVID-19 from CT scans (56), its clinical applicability in dental practice remains limited. The transition from perceiving AI as a threat to embracing it as a tool reflects its growing influence across healthcare (57–59). However, over-reliance on AI for diagnosis and treatment is discouraged, and dental professionals are urged to maintain oversight.

ChatGPT, a large language model developed by OpenAI, offers human-like text responses through ML and DL, enhancing computer communication and responsiveness. Its potential applications span education and patient care, particularly in providing information and managing expectations for pre- and post-surgical dental patients. ChatGPT can contribute to patient education, empowerment, decision-making, and service efficiency in dentistry. Lee et al. (60) discuss how large language models like ChatGPT can enhance medical education by providing interactive simulations and acting as virtual teaching assistants. While these models could improve student engagement, Jamal (61) emphasizes the need for more research to verify these benefits and highlights the importance of adapting curricula to keep pace with AI advancements. A framework for incorporating ChatGPT into medical education is also proposed, emphasizing both immediate enhancements in digital literacy and long-term strategies focusing on patient-centered care. In a study assessing ChatGPT's effectiveness, responses to 284 questions generated by 33 doctors across 17 specialties were found to be accurate and complete, indicating its potential as a reliable medical knowledge source (62). However, variability in performance suggests room for improvement. Another study (63) revealed that recent medical graduates are cautiously optimistic about using AI in their education and careers, with positive attitudes linked to prior AI experience. This underscores the need for formal AI education to prepare students for an AI-integrated healthcare environment. While ChatGPT and AI hold promise for transforming dental practice and education, challenges such as variability in performance, ethical concerns, and the need for clinician oversight remain critical to their effective implementation (64).

It is important to note that ChatGPT, like any other emerging technology, has its limitations. The system is operated by a vast neural network that is extremely computationally intensive and consumes a large amount of memory, making it prohibitively expensive for smaller medical practices with limited budgets (65). The model's inability to incorporate external data sources can negatively affect its accuracy, which is a critical factor in the medical field. Further, ChatGPT sometimes fails to cite references correctly or at all, and its responses may differ based on the context of the conversation, leading to convincingly incorrect responses (65–68). It is for these reasons that ChatGPT must properly express uncertainty to avoid misdirection. The advent of artificial intelligence technologies in dentistry, referred to as “Dentronics,” heralds a new phase of transformation with potential benefits, including improved reliability, reproducibility, precision, and efficiency. Additionally, Dentronics could enhance the understanding of disease mechanisms, risk assessments, diagnostic processes, and prognoses to improve patient outcomes (68). The integration of AI into dentistry continues to grow, however, it is unlikely to completely replace dentists, since the profession demands more than just disease diagnosis. It also involves integrating clinical observations and providing patient care. In spite of this, dental professionals would benefit from gaining a solid understanding of AI principles and methodologies as their field continues to evolve (69, 70).

## 4 AI in dental pathology, clinical practice, and surgery

Artificial intelligence (AI) has significantly transformed the medical and scientific landscapes, including its integration into various aspects of diagnosis, therapy, and patient care (71). The effectiveness of AI chatbots in providing accurate and trustworthy information is crucial for adoption in clinical decision-making. These tools facilitate discussions that may enhance diagnostic and treatment guidelines, particularly in dentistry. For instance, AI can help identify pathologies in the maxillary sinuses that may not be apparent in extraoral radiographs, thus reducing diagnostic errors for less experienced dentists. Kim et al. (72) demonstrated that AI can provide superior sensitivity and specificity compared to radiologists when analyzing Water's view radiographs. AI has also effectively identified conditions such as mucosal hypertrophy and retention cysts, which are sometimes overlooked by radiologists. Kuwana et al. (73) proposed a convolutional neural network (CNN) model to distinguish such conditions in CBCT images, highlighting AI's potential in improving diagnostic accuracy.

In oral cancer detection, early diagnosis is vital since it ranks as the sixth most prevalent cancer. Nayak et al. (74) demonstrated that AI using laser-induced autofluorescence spectra could accurately distinguish between normal, premalignant, and malignant tissues, suggesting its potential for real-time clinical use. AI's capabilities in predicting cancer recurrence and assessing lymph node involvement further improve patient care by offering accurate screening tools and personalized treatment approaches. ChatGPT, in particular, has shown promise in recognizing oral and maxillofacial disorders. Its ability to process large volumes of patient information can aid dental practitioners in diagnosis and treatment planning (75). With advancements such as GPT-4, which can interpret information from images, ChatGPT enhances diagnostic accuracy and assists in treatment guidance. However, while ChatGPT can provide timely and accurate information, it should not replace professional consultations, and further clinical validation is necessary.

A systematic review (76) highlighted ChatGPT's role in supporting diagnostic pathology. Despite challenges like the quality of training data and occurrences of “hallucinations,” the study noted ChatGPT's utility in providing extensive scientific information. However, it underscored AI's role as a supportive tool rather than a decision-maker. Concerns regarding the accuracy of AI-generated scientific references were highlighted in another study (77), which showed ChatGPT's low accuracy rate in generating reliable bibliographic information, emphasizing the need for integrating credible scientific databases. ChatGPT also has potential in dental research, as explored in a review (78), which showcased how its transformer-based architecture facilitates the efficient synthesis of data for systematic reviews. While the model streamlines research activities, it also presents challenges, such as limitations in understanding specific dental issues and potential biases in training datasets. The review emphasized the importance of integrating AI with human expertise to enhance research quality and outcomes.

In a study (21) conducted in Japan, deep learning was employed to detect fatty degeneration in salivary gland parenchyma, indicating Sjogren's syndrome. The study utilized 500 CT scans, with 400 for training and 100 for testing. The diagnostic performance of the DL system was on par with experienced radiologists and surpassed that of less experienced counterparts. As shown in Table 2, the followings are instances of the integration and application of digital dentistry in today's dental practices. Diagnosing salivary gland tumors is challenging due to their infrequent incidence and morphological similarities. Machine learning has been employed to discern malignant salivary gland tumors based on their cytological features. In a study by Chiesa-Estomba and colleagues (43), a comprehensive approach integrating clinical, radiological, histological, and cytological data was utilized to anticipate facial nerve dysfunction in patients undergoing surgical treatment for salivary gland tumors with potential posterior nerve injury. Artificial intelligence stands as a valuable diagnostic tool, offering the capability to predict facial nerve injury and providing proactive awareness for surgeons and patients about potential complications.

**Table 2:** Integration and applications of digital dentistry in modern dental practices.

Time Period	Application	Field	Critical technologies
Past	Digitized dental impressions	Dental reconstructions	Computer-aided design/manufacturing, Intra-oral digital scanning
Past	Assisted computer navigation for dental implants	Implantology	Cone beam computed tomography (CBCT), computer-aided Design/mnufacturing
Present	Three-dimensional dental replicas	Prosthetic dentistry, Orthodontic treatment	Three-dimensional fabrication, computer-aided design/manufacturing
Present	Machine learning detection of tooth decay	Diagnostic Procedures	Machine learning, digital image processing
Present	Virtual reality for dental education	Educational tools for patients	Virtual reality, Three-dimensional visualization
Future	Machine intelligence for treatment strategizing	Orthodontic planning, Maxillofacial surgery	Machine intelligence, digital image interpretation
Future	Biocompatible dental components produced by additive manufacturing	Dental implantology	Additive manufacturing, Computer-Aided Design/Manufacturing, Bioengineering materials
Future	Remote dental consultations via digital communication	Remote dental care	Telecommunication, Digital communication platforms, diagnostic imaging technology
Future	Tailored dental prosthodontics	Prosthodontics	Digital dental mapping, Computer-Aided Design/Manufacturing, Three-dimensional fabrication
Future	Utilization of advanced language models in dentistry	Dental diagnosis automation and multimodal diagnostic evaluation	Advanced linguistic computational models, specifically ChatGPT
Future	Customized conversational assistance	Patient Consultation	Conversational AI, Chatbot technology
Future/present	Preparing prospective dental professionals	Dental undergraduate education	Augmented Reality (AR), Virtual Reality (VR)
Present	Advancement and evaluation of color matching in dental restorations	Dental restoration color matching	Traditional methods versus spectrophotometric benchmarking
Present	Transition to digital radiography	Routine dental operations	Enhancement of image luminance and contrast, specialized image processing algorithms, sensor-agnostic technologies

The incorporation of Artificial Intelligence (AI), particularly ChatGPT, into clinical dental practice and surgery represents a paradigm shift, promising to enhance diagnostic capabilities, improve surgical precision, and revolutionize patient care. In a study (79), researchers evaluated ChatGPT's utility in dental education and its potential as a clinical decision support system. The study gathered insights from 27 specialists in nine dental fields and assessed ChatGPT's responses to 243 dental questions. Oral medicine and radiology received the highest ratings, indicating a general precision in ChatGPT's answers. However, the study also emphasized the importance of human oversight, cautioning against replacing professional consultations with AI. Another study (80) discussed ChatGPT's role in surgical training, specifically in otolaryngology, emphasizing its value for educational purposes during periods of reduced clinical exposure, such as the COVID-19 pandemic. The authors highlighted ChatGPT's ability to enhance learning by simulating patient communication and providing tailored responses. A study (81) compared ChatGPT's recommendations for laryngology and head and neck surgery cases with those of board-certified otolaryngologists. While ChatGPT accurately provided primary diagnoses and treatment options, it tended to suggest more tests than necessary, highlighting its potential as a supplementary diagnostic resource.

**Figure 4:**
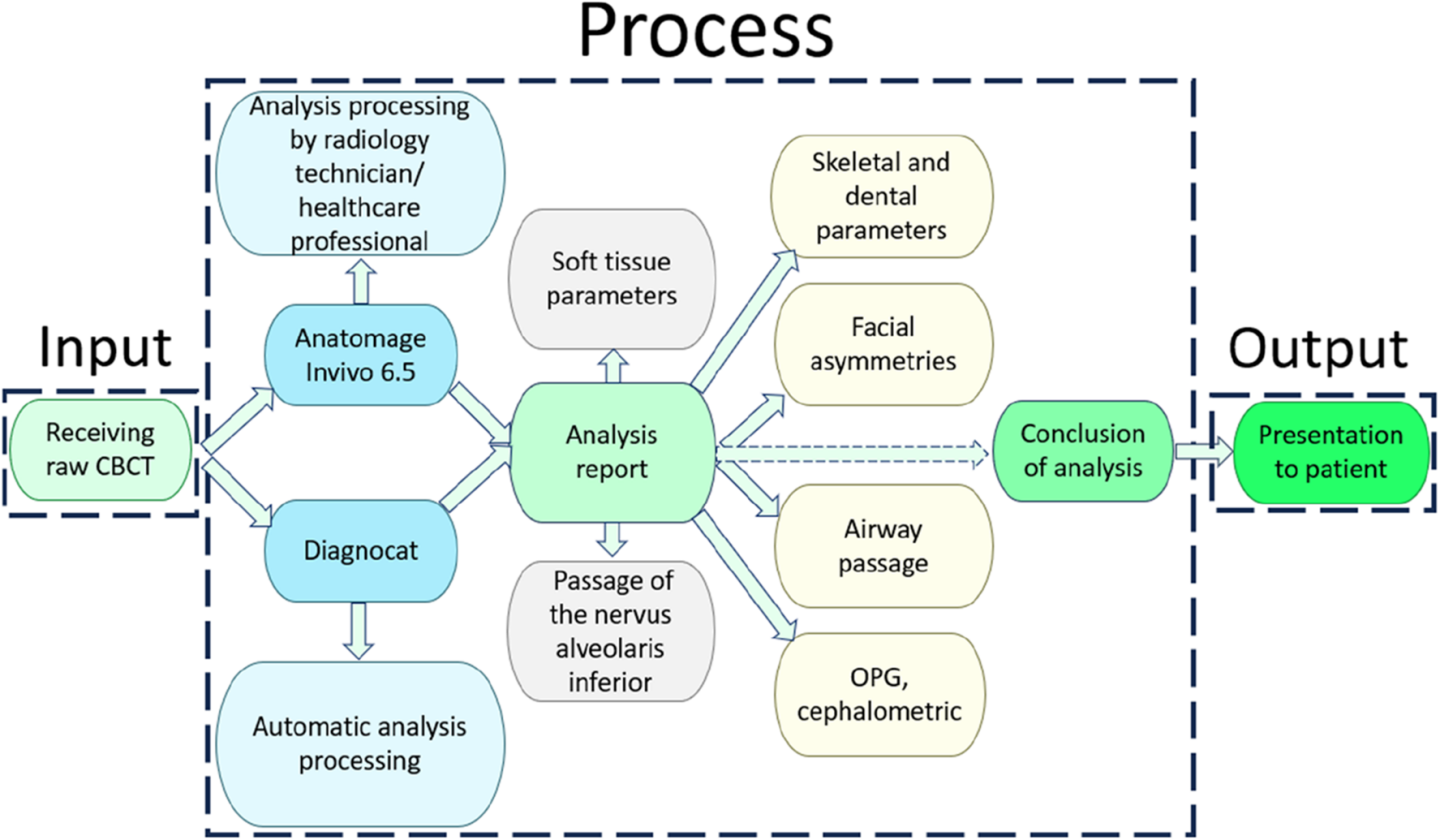
Workflow for processing and segmenting CBCT data for patient presentation.

Thorat et al. (82) explored the impact of ChatGPT on undergraduate dental education, particularly focusing on its ability to support personalized learning, evidence-based assessment, and integration into virtual simulations. ChatGPT was recognized for enhancing learning diversity and offering multilingual support, making it a valuable tool for modern dental education. According to Saravanan's study (83), the use of ChatGPT in oral and maxillofacial surgery was evaluated in terms of its impact on patient care, surgical precision, and integration into traditional practices. The study emphasized the potential benefits of ChatGPT in enhancing patient communication, improving surgical outcomes, and supporting education, while also highlighting the need for a balanced approach between AI and traditional methods. The integration of ChatGPT into dental practice also includes enhancing communication between patients and healthcare providers. Figure 4 illustrates the workflow for processing Cone Beam Computed Tomography (CBCT) data and presenting it to patients. Within this workflow, ChatGPT can enhance patient comprehension by automating the generation of interpretive reports, distilling complex diagnostic data into accessible summaries, and translating technical jargon into clear language—ultimately empowering patients to better understand their scan results.

**Figure 5:**
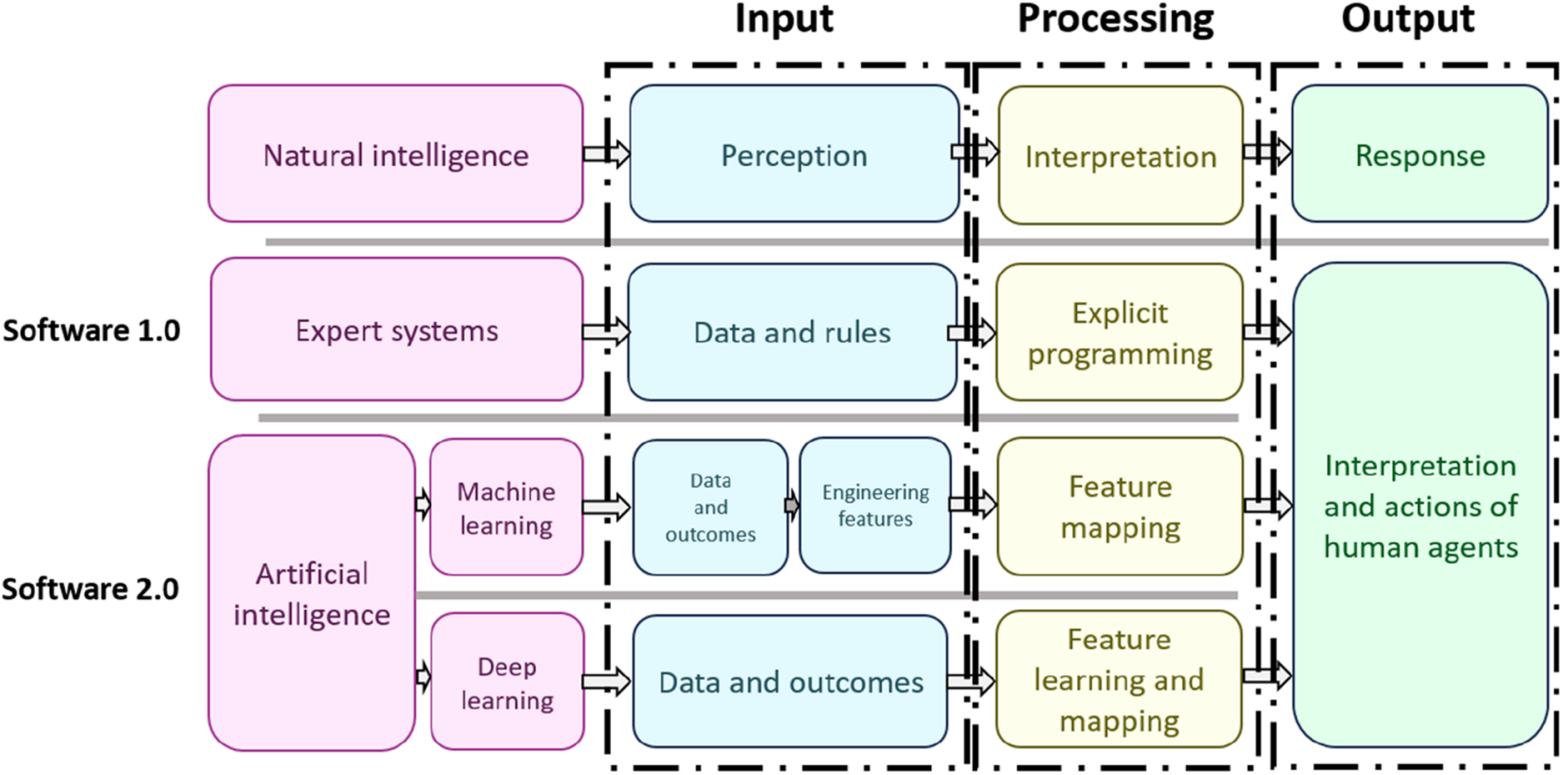
An illustration of the evolution of artificial intelligence from software 1.0, which consisted of expert systems that used explicit programming to interpret user input, to software 2.0, which incorporates advanced methods such as machine learning and deep learning.

The ChatGPT application can also provide personalized explanations of the treatment implications based on the diagnostic conclusions, thus bridging the gap between technical medical findings and patient education. As a result, patients are more informed and engaged, which is crucial for successful healthcare outcomes. A study conducted by Shan et al. (84) underscores that AI serves as a complementary tool for dental professionals rather than a substitute. It also emphasizes the current advantages of AI in improving diagnostic accuracy, aiding in treatment planning, and predicting outcomes. Moreover, they mention the challenges associated with AI's broader adoption, such as data management, algorithm transparency, computational demands, and ethical implications. The authors suggest a collaborative approach to integrate AI into dentistry, suggesting that AI can improve efficiency and outcomes, but cautioning that challenges such as methodological limitations, data scarcity, and ethical concerns must be addressed, with human oversight and evidence-based practices being crucial to maintaining trust in AI applications in dentistry. The review by (85) also highlights the significance of ‘explainable AI’ to demystify AI processes for users and the necessity of updating dental education to include digital proficiency to keep up with the pace of technological advancement. Li et al. (86) discuss GPT-4's potential in neurosurgery, including its contribution to brain-computer interfaces and understanding neural behaviors, offering insights into therapeutic approaches for neurological conditions. Figure 5 in the study, illustrates the progression from early expert systems to contemporary AI in software development, emphasizing their use in clinical dental practice and surgery. Traditional intelligence follows a linear path from perception to interpretation and then to response, mirroring human cognitive function. “Software 1.0” represents early expert systems that mimic human cognition by applying predefined data and rules through explicit programming, but these systems require human interpretation of their outputs. “Software 2.0”, encompassing machine learning and deep learning, represents a more advanced phase of AI where systems learn from data outcomes. In machine learning, experts design features to guide algorithms, which are then mapped to outputs (87).

Deep learning automates the learning process from raw data, enabling more complex data processing and outputs that mimic autonomous human responses. The rapid growth of AI in healthcare, fueled by advancements in computational power and data availability (88), is particularly evident in oral and maxillofacial surgery (OMFS), where convolutional neural networks are increasingly used for image-based diagnostics.

AI applications in dentistry are transforming diagnosis and treatment, offering unprecedented precision, efficiency, and personalized care. He et al. (89) discuss the utility of ChatGPT and GPT-4 in healthcare, highlighting their potential benefits for surgical planning and patient communication, but caution that these tools should be used under supervision due to their lack of nuanced clinical expertise. While GPT-4 can handle large datasets and act as a virtual assistant across medical disciplines, it remains supplementary to a physician's judgment. Balel et al. (90) recognize ChatGPT's usefulness in disseminating patient information within oral and maxillofacial surgery, recommending its integration into clinical practice with caution. Ferres et al. (91) further suggest ChatGPT's ability to amalgamate diverse data types could enhance radiology by combining images, text, and patient records for tasks like report generation.

Hiroj Bagde et al. (92) conducted a systematic review and meta-analysis to evaluate ChatGPT's accuracy in medical and dental fields. Although ChatGPT's accuracy ranged from 18.3% to 100% across different studies, it generally performed better than chance, suggesting its potential as a research tool in dentistry and medicine. However, caution is advised, and further research is needed to enhance its dependability. Another study (45) evaluated ChatGPT-4's responses to 30 oral surgery-related questions, finding a 71.7% accuracy rate, as assessed by an expert oral surgeon. This highlights ChatGPT-4's potential as a supplementary clinical decision-making tool in dentistry, while emphasizing it cannot replace the expertise of a qualified oral surgeon. The study calls for further research to ensure safe and effective use of AI technologies like ChatGPT-4 in dental specialties.

Chau et al. (93) evaluated ChatGPT versions 3.5 and 4.0 against dental licensing exams from the US and UK. ChatGPT 4.0 showed improved performance, accurately answering 80.7% and 62.7% of questions from the UK and US exams, respectively, and even passing both exams. This marked improvement over ChatGPT 3.5 highlights the potential of Generative Artificial Intelligence in dental education and professional development, though the study calls for further advancements in GenAI. A study by Rizwan and Sadiq (94) assessed ChatGPT's capability in diagnosing and managing cardiovascular diseases (CVDs) through ten hypothetical clinical cases. With a successful diagnosis rate in eight cases, ChatGPT's responses, verified by cardiologists, aligned with current medical guidelines. Despite its limitations in providing specific treatment plans, ChatGPT was found to be a useful tool for medical professionals, particularly for junior doctors, in formulating diagnosis and treatment strategies, highlighting the importance of complementing AI with expert clinical judgment. Batra et al. (95) explored AI's transformative impact on the dental industry, focusing on its potential to enhance diagnostic and treatment accuracy. The study addresses AI's application in various dental care aspects, including sophisticated imaging, disease detection, and the creation of personalized treatment plans. It also considers ethical and privacy concerns related to AI in dentistry, envisioning a future where AI-integrated dental practices lead to more efficient, patient-centered, and effective oral health outcomes, emphasizing the shift towards leveraging technology to improve the quality of patient care. According to Cheng et al. (96) and Hassam et al. (97), GPT-4 can enhance clinical support for spinal surgeons and joint arthroplasty by creating AI-based virtual assistants. A study by Rao et al. (98) demonstrated ChatGPT's utility in radiologic decision-making and clinical support, while other research has demonstrated its effectiveness in providing dental hygiene and health information to patients. The results of these studies demonstrate that ChatGPT and GPT-4 are becoming increasingly important as supportive tools in healthcare, enhancing professional practice and patient care. There are several studies that demonstrate the benefit of artificial intelligence in diagnostics, therapeutic decisions, surgical planning, and prognosis (99). AI is capable of enhancing learning, classification, prediction, and detection, thus potentially reducing human error and augmenting the skills of clinicians. Furthermore, they highlighted the need for AI algorithms to undergo rigorous clinical validation and ethical scrutiny regarding data protection (100–102). The versatility of AI in dentistry is highlighted in numerous studies, demonstrating its capability to enhance diagnosis, treatment planning, and patient outcomes. To provide a clearer understanding of the breadth of AI applications in dental specialties, Table 3 offers a survey of various studies that explore these developments.

**Table 3:** A survey of AI applications in dentistry.

Authors	Year	Aim	Method	Result
Alzaid et al. (21)	2023	To provide an overview of AI applications in various dental specialties	Exhaustive literature search on AI applications in dentistry	Highlighted AI's role in improving disease diagnosis and treatment planning, though AI cannot replace dentists
Tiwari et al. (18)	2023	Investigate ChatGPT's applications in public dental health for research, education, and clinical practice	Systematic literature review on ChatGPT's implications in public health dentistry	ChatGPT aids in scientific writing and research, with potential benefits and risks noted
Khurana, and Vaddi (103)	2023	Explore the limitations and potential applications of ChatGPT in academic oral and maxillofacial radiology (OMFR)	Editorial overview based on authors’ experiences and relevant literature	Identified valuable applications in education and limitations in image-based question answering and authorship validity
Alhaidry et al. (66)	2023	Discuss the use of ChatGPT in dentistry for diagnoses, disease risk assessment, and other applications.	Review of literature on ChatGPT's use in dentistry.	ChatGPT benefits include anomaly detection and workload reduction, but with notable risks and limitations.
Bagheri and Varzaneh (104)	2023	Review applications of ChatGPT and GPT4 in biology, medical and dental studies, and health care, along with concerns about its use	Article review focusing on ChatGPT and GPT-4's contributions and limitations in science and healthcare	Highlighted ChatGPT's impact on various fields despite limitations and raised concerns on potential misuse
Zhou et al. (105)	2023	Examine how AI can transform dentistry, improve patient outcomes, and streamline tasks, with a focus on ethical, legal, and regulatory implications	Analysis of AI applications in dentistry, particularly in diagnosis and treatment planning	AI improves diagnosis accuracy and operational efficiency but requires careful management of over-reliance and ethics
Kavadella et al. (106)	2024	Evaluate ChatGPT's implementation in dental education quantitatively and qualitatively	Mixed methods study with 77 dental students using ChatGPT for a learning assignment	ChatGPT group performed better in knowledge exams; students recognized its benefits and limitations
Kamath et al. (107)	2024	Highlight AI's role in dentistry and predict its future impacts	Narrative review of 59 papers from Google Scholar and PubMed	AI useful in various dental phases; future advancements anticipated in dental tools and education
Büttner et al (108)	2023	Summarize NLP applications and limitations in dentistry.	Narrative review	NLP has potential for applications in dentistry, though challenges remain in its broader adoption
Younis et al. (109)	2024	Explore AI's transformative potential in healthcare, including dentistry	Systematic literature review and meta-analysis of 82 papers	ChatGPT and AI tools show promise in medicine and healthcare for various applications, with ongoing challenges
Ali et al. (110)	2023	Evaluate ChatGPT’s performance in dental education assessments	Exploratory study on ChatGPT’s accuracy in dental curricula assessments	ChatGPT provided accurate responses to most assessments, indicating potential to revolutionize virtual learning and assessments
Bragazzi et al. (99)	2023	Assess ChatGPT’s diagnostic accuracy in endodontics	Analysis of 70 peri-apical X-rays by ChatGPT	ChatGPT showed limited clinical usability in current form, with a need for improvements in dental diagnostics accuracy

**Table 4:** An overview of existing medical LLMs and their model development.

Domains	Model development archetypes	Scale of parameters	Data magnitude
Pre-training	B-ERT Large	Extensive	Large-scale data
	PubM-ERT Large	Medium	Considerable corpus
	ClinicalB-ERT	Medium	Broad clinical notes
	MedC-ERT	Medium	Ample scientific articles
	BioM-ELMo	Small	Large data repository
	OphthGPT	Medium	Substantial dialogues
	GatorTron Large	Medium	Large token database
	GatorTron GP	Small	Moderate token database
	MEDTRON	Small	Extensive notes
	DoctorLM	Medium	Vast dialogues
	BioQuest	Medium	Extensive dialogues
	ClinicalGPT	Small	Wide EHRs and QA
	QliM-Med	Small	Broad dialogues
Medical-domain LMs	CharDoctor	Small	Focused instructions
	BenTaa	Small	Specific instructions
	HuatoGP	Small	Extensive instructions and dialogues
	Baize-healthcare	Medium	Broad dialogues
	MedAePac	Small/Medium	Focused medical QA
	AlpCure	Small	Specific instructions
	Zhiyin	Small	Dedicated instructions
	PMCLLM	Medium	Extensive tokens
Fine-tuning	CPLLM	Medium	EHRs
	Clinical Camel	Small/Medium	Extensive articles and QA
	MedLP-M	Medium	Focused medical QA
	BioPump	Medium	Extensive tokens
	CoeDP	Medium	Extensive thought pieces
	CadX	Small	Specific instructions
	Dr. Kumo	Small	Zero-shot learning
	CharPZ	Small	Zero-shot learning
	MedPrompt	Medium	Dedicated prompting
	MedPromptM	Medium	Few-shot learning

The medical field often emphasizes algorithmic thinking, but healthcare providers sometimes resort to heuristic decision-making due to heavy workloads and cognitive demands, which can lead to errors due to factors such as experience, emotional state, fatigue, and personal characteristics. AI is rapidly being developed in healthcare and is praised for its ability to support medical decision-making processes and minimize cognitive biases and errors. According to the studies, AI methodologies may be beneficial at all stages of patient care, from initial screening to recovery post-surgery (111).

## 5 An exploration of the capabilities of LLMs in dentistry

LLMs can automate the comprehension of documents and enable the analysis of treatment plans, leveraging the advantages of extensive pretraining. Additionally, exposure to billions of documents helps LLMs develop natural language reasoning (NLR) capabilities for context understanding. This NLR capability can enhance the efficiency of dental practitioners in crafting personalized treatment plans based on patients’ backgrounds. For example, NLR algorithms can scrutinize patterns of adverse drug reactions (ADRs) associated with various dental procedures and medications. Understanding common ADRs linked to drugs allows dentists to adjust their treatment plans, reducing the likelihood of side effects such as gum bleeding and severe conditions like bisphosphonate-related osteonecrosis. Alexander Fuchs et al. (112) evaluated the effectiveness of ChatGPT versions 3 and 4 by employing questions from the European Examination in Allergy and Clinical Immunology (EEAACI) and the Swiss Federal Licensing Examination in Dental Medicine (SFLEDM). They explored the effect of priming on enhancing ChatGPT's responses. ChatGPT 4 notably outperformed version 3 in all tests, with priming improving its performance, especially for SFLEDM questions. This study illustrates the rapid advancement in large language model technology and the potential benefits of priming yet cautions against the uncritical application of such models in healthcare due to inherent risks and limitations. A study (113) at Meharry Medical College examined ChatGPT's integration into the dental curriculum, highlighting both its potential and challenges in enhancing dental education. The research analyzed the chatbot's responses to various queries using course materials, showcasing ChatGPT's utility in assisting with academic writing and content creation. Despite identifying limitations, the study emphasizes the importance of rigorous evaluation and the combination of AI technologies like ChatGPT in dental education to improve teaching and learning quality.

A comparative study (29) evaluated the performance of GPT-3.5, GPT-4, and Google Bard using the Japanese National Dentist Examination (JNDE) to assess their clinical application potential in Japan. GPT-4 led in overall correctness, with Bard excelling in essential questions, indicating the distinct capabilities and possible clinical uses of these models in dentistry. The findings suggest a promising future for LLMs in clinical settings, though further validation is needed to ascertain their global utility. Koubaa et al. (114) provided a review of ChatGPT, analyzing its technological advancements and the breadth of research surrounding it. This first extensive literature assessment on ChatGPT outlines future research challenges and directions, offering valuable insights for stakeholders interested in the model's applications, implications, and development prospects. Ana et al. (115) critically appraised studies observing the connection between dentofacial features and dental trauma among Brazilian children and adolescents, focusing on the handling of confounding factors. Despite some acknowledgment of biases, the review found a general lack of thorough consideration for confounders in these studies, underscoring the difficulty in establishing causality from such research. This highlights the need for more rigorous methodological approaches in observational studies within the field.

**Figure 6:**
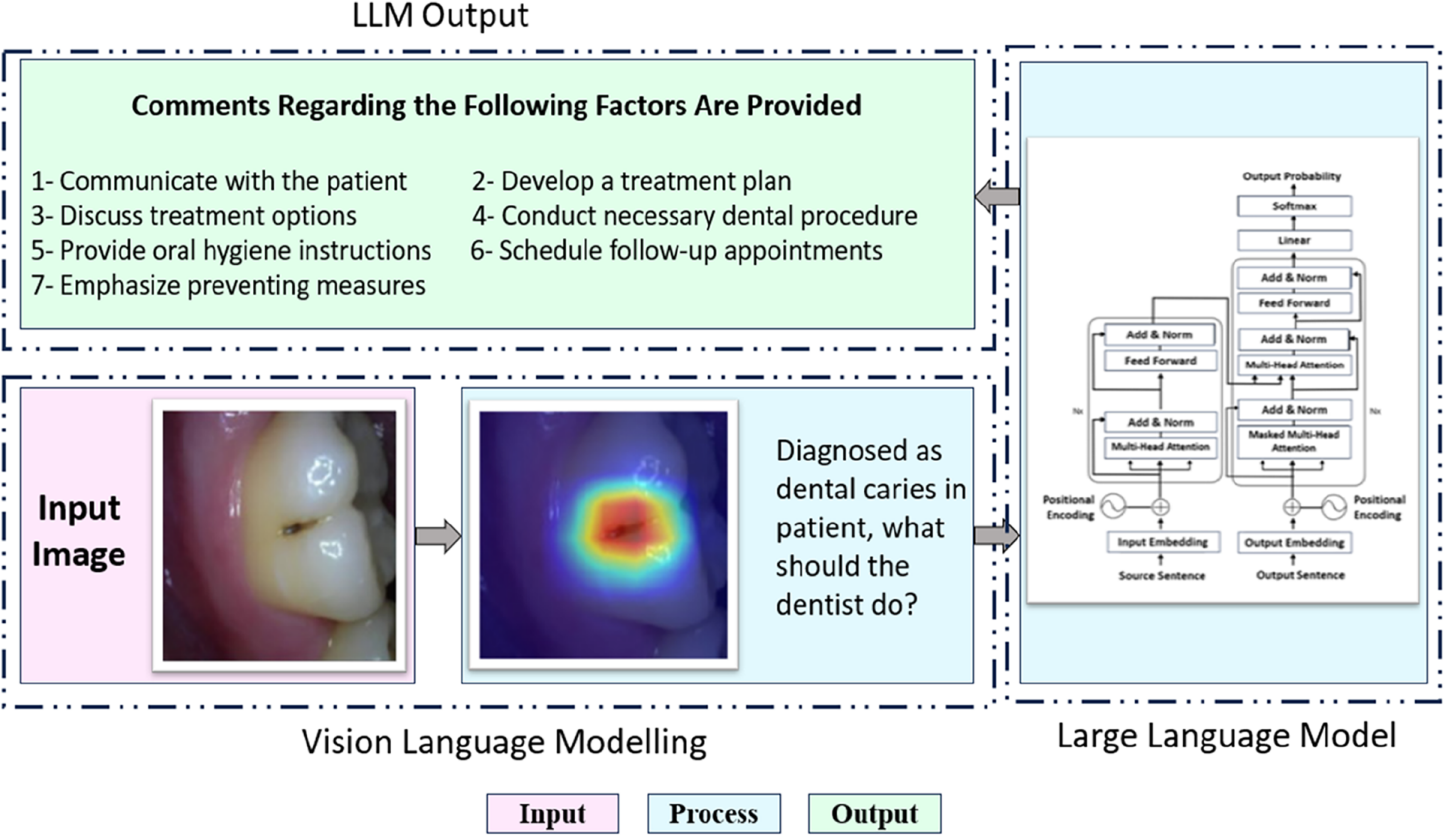
Process of large language model (LLM) for dental care.

The goal of Supervised Fine-Tuning (SFT) is to make use of high-quality medical corpora, such as knowledge graphs (116), physician-patient discussions (117), and medical question-answering (118). In order to further pre-train the generic LLMs with the same training objectives, such as next token prediction, the produced SFT data acts as a continuation of the pre-training data. Through the additional pre-training period offered by SFT, general LLMs can become more knowledgeable about medicine and more in line with the medical field, enabling them to become specialized medical LLMs. Supervised fine-tuning (SFT), Instruction fine-tuning (IFT), and parameter-efficient tuning are some of the methods used nowadays for fine-tuning (119). Table 1 provides an overview of the refined medical LLMs that were produced. The ChatGPT AI model utilized in this study is text-based, hence it cannot directly read radiologic images. Consequently, the final diagnoses were produced using descriptive radiologic results (text data). Future studies could benefit from using AI models for picture segmentation and captioning to generate radiologic findings that are descriptive. ChatGPT can then use these findings as the foundation for further diagnostic conclusions (120). The process of describing an image's visual information in natural language using a visual understanding system and a language model that can produce coherent, syntactically accurate sentences is known as image captioning (121). In addition, the newly launched ChatGPT4V now supports the entry of photos in addition to text. Writing radiological reports could undergo more alterations as a result of all these AI models. Figure 6 illustrates a workflow in which an LLM, integrated with vision language modeling capabilities, processes an input image of a dental condition such as caries and generates a multifaceted output tailored for dental practices.

The LLM analyzes the input image to understand the dental issue and generates an action plan for the dentist to follow, illustrating how artificial intelligence can assist with clinical decision-making. Figure 6 illustrates how an LLM can streamline this analysis (122). By reviewing patient histories, NLR can also identify comorbidities, monitor drug safety, and enhance patient education through natural language generation-based medical documentation utilizes high-quality medical corpora, including physician-patient dialogues (123), medical Q&A (124), and knowledge graphs (116, 125), to continue the pretraining of general LLMs with the same objectives, such as predicting the next word or phrase. This additional pretraining phase enables the LLMs to acquire extensive medical knowledge and become attuned to the medical field, effectively transforming them into specialized medical LLMs. Fine-tuning techniques like SFT, Instruction Fine-Tuning (IFT), and Parameter-Efficient Tuning refine these models further. The findings suggest that dentists could be supported in providing thorough, informed care by a sophisticated artificial intelligence system.

## 6 Conclusion

In conclusion, while AI technologies like ChatGPT have demonstrated promising potential in the field of dentistry, their integration into clinical practice should be approached with significant caution. The advancements in Large Language Models (LLMs) have enabled systems like ChatGPT to assist with tasks such as patient education, diagnostics, and research support. However, this study strongly emphasizes that the current capabilities of AI are far from sufficient to replace the expertise of trained dental professionals. Future research must involve a more diverse and extensive pool of dentists to critically evaluate AI's role in dentistry and its ability to generate reliable, nuanced information. A key concern is the comparison between ChatGPT's output and that of dental students or postgraduates, which may reveal significant gaps in AI's understanding of complex, case-specific nuances. While ChatGPT can provide general information and streamline administrative tasks, it cannot replicate the depth of clinical judgment required for personalized treatment. This limitation becomes particularly concerning when considering the variability in patient cases, where AI may oversimplify conditions or fail to account for subtle clinical indicators.

Moreover, the over-reliance on AI systems like ChatGPT in dentistry could pose risks to patient safety. Despite the tool's ability to assist in education or initial diagnostics, AI-generated advice should not be blindly trusted or treated as definitive. There is a real danger that patients, or even inexperienced practitioners, might place too much confidence in these systems, leading to misdiagnoses or inadequate treatment plans. ChatGPT's reliance on pre-existing data and inability to understand real-time clinical contexts underscores its current limitations. The paper urges dental professionals to recognize that while AI can play a supportive role, it should not undermine the value of human expertise. Dentistry involves much more than diagnosis. It requires understanding individual patient needs, providing personalized care, and exercising clinical intuition built through years of experience. The risks associated with overestimating ChatGPT's capabilities must not be ignored, as errors in diagnosis or treatment planning could have severe implications for patient health.

Therefore, the future of AI in dentistry must focus on responsible integration, ensuring that AI tools like ChatGPT are utilized only as supplementary aids rather than primary decision-makers. Further research must rigorously assess these technologies to safeguard patient outcomes and ensure that human expertise remains central to the practice of dentistry. AI, while a revolutionary tool, must be handled with care, and its use must always be accompanied by a human-centered approach to clinical decision-making and treatment.

### 6.1 Limitation

ChatGPT offers considerable promise, but it is still in the early stages of development. The use of ChatGPT for information comes with ethical considerations, and it cannot be claimed as a doctor. However, it can be a valuable clinical tool. The human intellect remains irreplaceable by ChatGPT. To ensure that the model is accurate and reliable before its application in clinical settings, it is essential to verify its accuracy and reliability. To overcome the limitations of the ChatGPT model, it is necessary to continuously update and enhance it, considering user feedback. For healthcare professionals and patients, it is important to establish clear protocols regarding the appropriate use of ChatGPT. Furthermore, policies must be in place to protect patient confidentiality, and ethical standards must be established to navigate the complex issues presented by the integration of ChatGPT into healthcare.
